# Early Neutrophil Responses to Chemical Carcinogenesis Shape Long-Term Lung Cancer Susceptibility

**DOI:** 10.1016/j.isci.2020.101277

**Published:** 2020-06-17

**Authors:** Stefanie K. Wculek, Victoria L. Bridgeman, Freddie Peakman, Ilaria Malanchi

**Affiliations:** 1 Tumour Host Interaction Laboratory, The Francis Crick Institute, 1 Midland Road, NW1 1AT London, UK

**Keywords:** Immunology, Immune System Evolution, Cancer

## Abstract

Neoplastic transformation causing cancer is a key problem in tumor biology and can be triggered by exposure to environmental substances. We investigated whether the cellular composition of a tissue contributes to its predisposition to cancer upon a specific carcinogen. Neutrophils are important immune components involved in cancer progression, but their contribution to generation of transformed cells is elusive. Yet, neutrophil-released reactive oxygen species (ROS) can cause tissue damage, which potentially favors tumorigenesis. Here, we show that neutrophils contribute directly to neoplastic transformation by amplifying the genotoxicity of urethane in lung cells via ROS. Neutrophil-driven ROS-dependent DNA damage is timely restricted to urethane exposure and notably uncoupled from broad tissue damage or inflammation. Neutropenic granulocyte colony-stimulating factor (Gcsf)-knockout mice show reduced lung tumorigenesis, and forcing neutrophil recruitment only during urethane exposure rescues cancer incidence months later. This study shows that the time-restricted neutrophil response to carcinogens can impact the long-term tissue susceptibility to cancer.

## Highlights

• Neutrophils amplify urethane-induced DNA damage in the lung during cancer initiation
• Lung neutrophil-derived ROS drive urethane genotoxicity, but not tissue damage
• Neutropenic GCSF-ko mice show reduced urethane-induced lung carcinogenesis
• Forced early neutrophil mobilization rescues carcinogenesis in GCSF-ko mice

## Graphical Abstract

**Figure:**
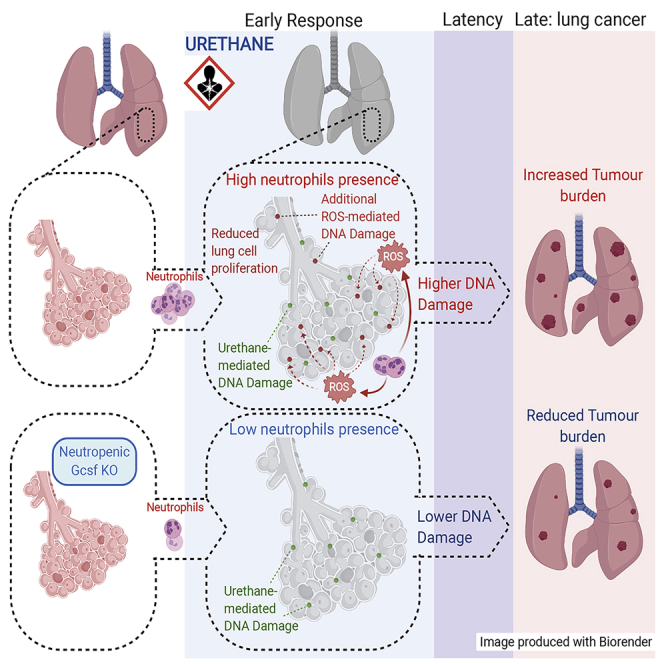

## Introduction

The strong impact of environmental factors as key determinants of cancer development was highlighted by epidemiologic studies showing that people who migrated to distant countries developed cancer types typical of the local population rather than of their homelands (Higginson et al., 1992). Numerous chemical carcinogens, including cigarette smoke, can trigger genetic mutations that are at the origin of cancer. Crucially, genotoxic events inducing tumorigenesis are accompanied by a promoting inflammation (Coussens and Werb, 2002). Particularly, the important role of neutrophils, the first inflammatory cells to be recruited to the affected tissue upon injury, in patients with cancer, is progressively becoming evident (Shaul and Fridlender, 2019). Notably, neutrophils are emerging as an important player not only in tissue injury but also in post-injury tissue regeneration (Phillipson and Kubes, 2019). Several recent studies have shown conflicting functions of neutrophils in both promoting and limiting tumorigeneses (Coffelt et al., 2016), suggesting a context-dependent regulation.

The potential of neutrophils to induce oxidative DNA damage *ex vivo* on epithelial cells was long recognized (Knaapen et al., 1999), and more recently, in the context of chemically induced intestinal tumorigenesis, neutrophils were reported to trigger genome-wide oxidative DNA damage in the epithelium and to contribute to mutations and cancer (Canli et al., 2017). Notably, this neutrophil activity was observed in the context of an acute inflammatory response where the tissue-damaging function occurs while blocking potential bacterial infection. However, in tissue where neutrophils are normally present to maintain homeostatic conditions, their tissue-damaging behavior needs to be tightly contained. Therefore, whether neutrophils directly contribute to the organ predisposition to cancer in the absence of an acute inflammatory response is currently unknown.

Here, using a model of lung cancer caused by the exposure to the genotoxic chemical urethane, we show that neutrophil reactions change the early tissue response to the chemical oncogenic stimuli in the absence of a broad inflammatory response. This neutrophil response amplifying DNA damage in adjacent cells is able to modify long-term tumor outcome. Therefore, we here show that tumor onset results from a whole-tissue response to the oncogenic stimuli, and that excluding even a single cellular component is sufficient to twist the final outcome.

## Results

### Neutrophils Show an Early Lung Recruitment Response upon Urethane Administration, and Their Presence Is Required for Efficient Lung Carcinogenesis

**Figure 1:**
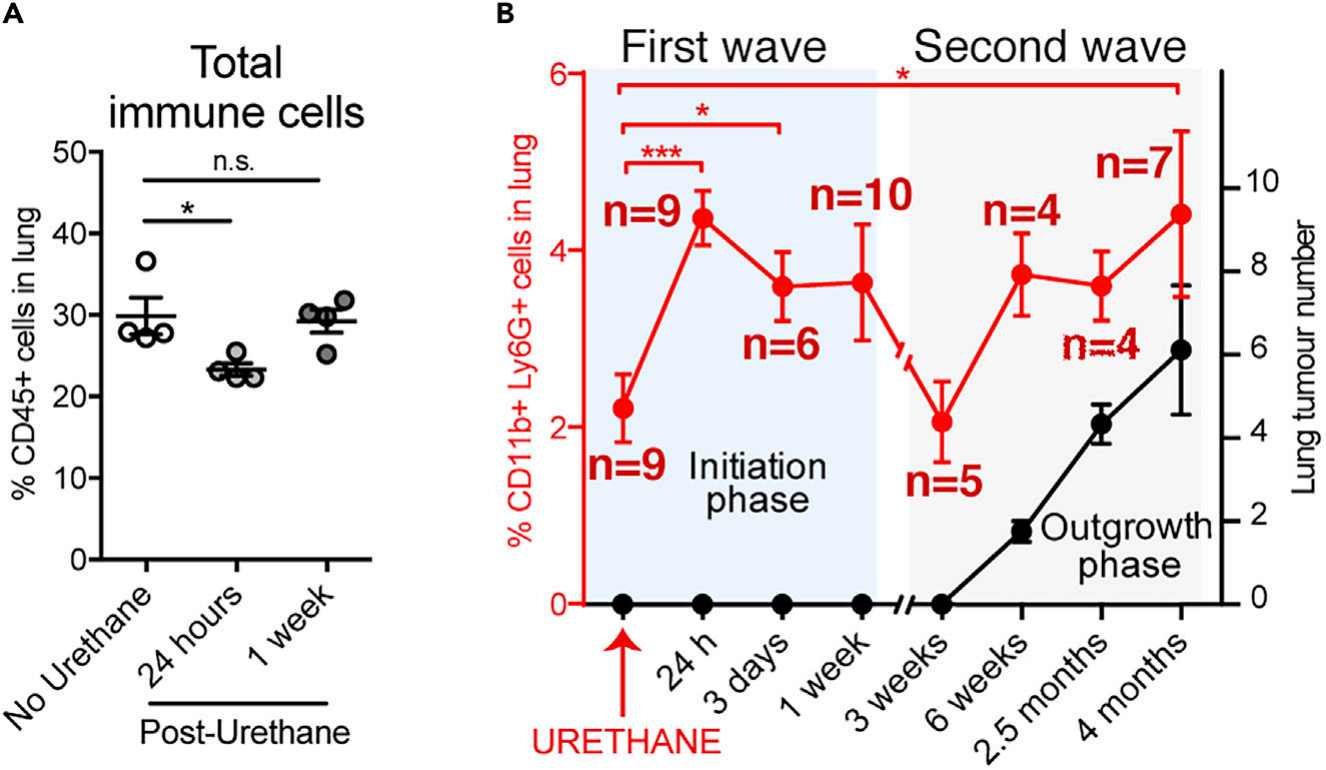
Carcinogen Treatment Induces Rapid Lung Infiltration of Neutrophils (A) Flow cytometric quantification of frequency of CD45+ total immune cells in the lung of wild-type mice at indicated times after urethane treatment. Data are represented as individual values and mean ± SEM (n = 4 per time point), ∗p < 0.05 (Student's t test); n.s., not significant. (B) Time course analysis of the lung of wild-type mice after urethane treatment at indicated times. Flow cytometric quantification of CD11b+ Ly6G+ neutrophil frequencies in the lung (left y axis, red curve and red stars) and macroscopic quantification of surface lung tumor number (right y axis, black curve). Blue-shaded time frame (first wave of neutrophil infiltration) represents the initiation phase of urethane-mediated carcinogenesis, and red-shaded time frame (second wave) represents the tumor outgrowth phase. Data are represented as mean ± SEM (n ≥ 4 per time point), ∗p < 0.05, ∗∗∗p < 0.001 (Student's t test). See also Figure S1.

Cancer is essentially a genetic disease (Vogelstein and Kinzler, 2004), and the first driver of tumor initiation is the generation of oncogenic mutations. Urethane is a potent genotoxic agent of cigarette smoke and generates the complex mutation spectra of human cancers in mice (Westcott et al., 2015). The urethane lung cancer model involves one acute exposure to urethane via intra-peritoneal injection, which, several weeks later, results in lung cancer onset without inducing a prominent tissue damage response. Interestingly, we observed a short-term reduction in overall immune cell presence in the lung upon urethane exposure (Figure 1A), which reflected a dynamic remodeling of the CD45+ immune cell compartment (Figures S1A–S1F). Presence of T and natural killer (NK) cells is temporally reduced in lungs 24 h after urethane treatment and recovers after 1 week (Figures S1A and S1B). Myeloid cell populations, such as dendritic cells and alveolar macrophages, are also notably lost 1 week post-urethane (Figures S1D and S1E). In notable contrast, neutrophils increased their presence in the lung tissue 24 h after urethane injection (Figure 1B). Their amount is normalized by the end of the first week, and a second increase can be observed concomitantly with lung tumor growth, approximately 6 weeks post urethane treatment, and persists during the entire cancer outgrowth phase (Figure 1B). Hence, neutrophils appear to have two waves of response: very early after the genotoxic insult, during occurrence of genetic mutations, and later on, when competent neoplastic cells drive tumor outgrowth.

**Figure 2:**
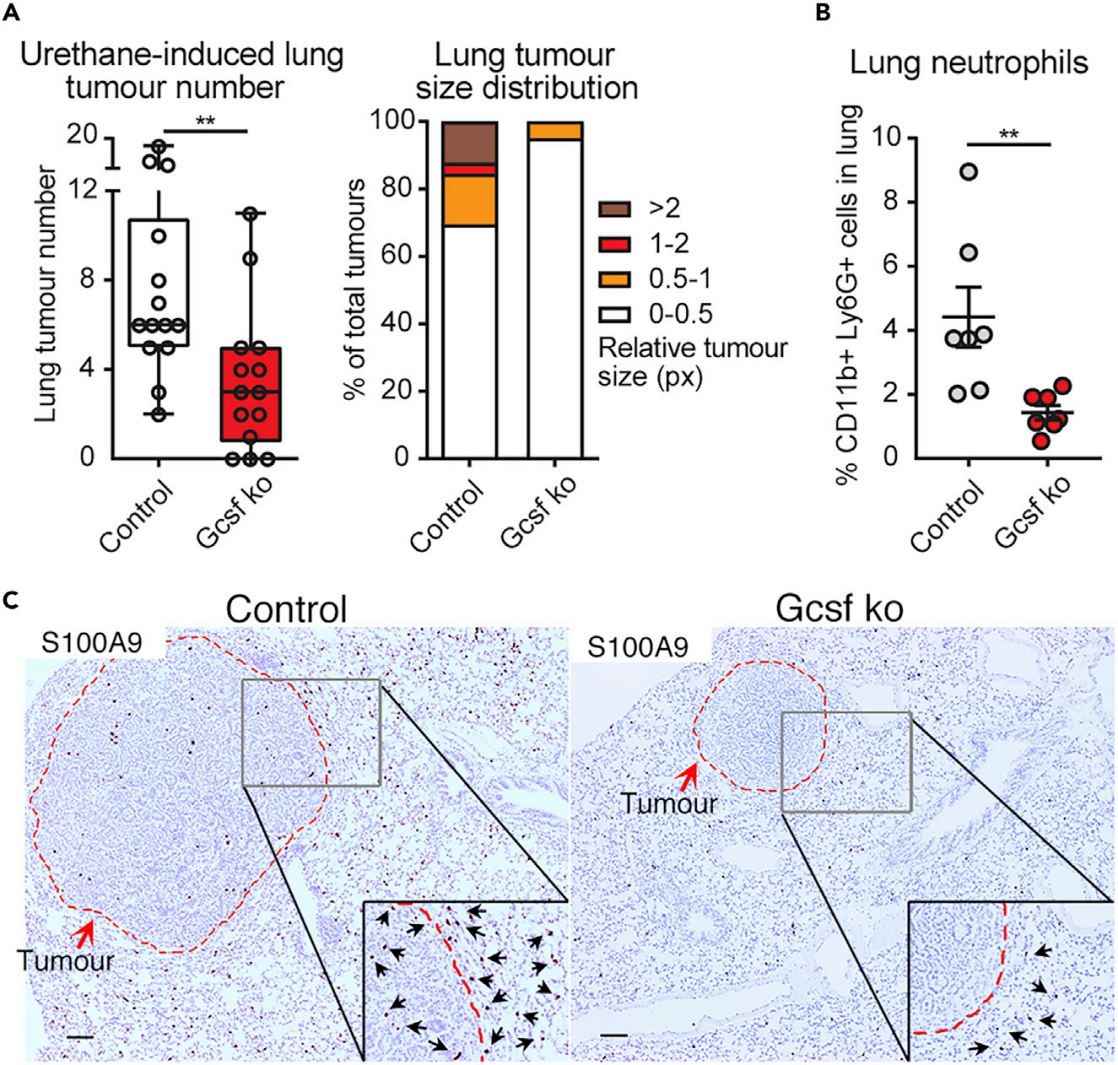
Gcsf ko Mice Are Less Susceptible to Chemical Carcinogen-Induced Lung Tumorigenesis Csf3 +/+ or +/− (Control) mice and Csf3 −/− (Gcsf ko) littermates were treated with urethane and lungs analyzed 4 months later. (A) (Left panel) Macroscopic quantification of visible lung tumor number by counting nodules on the surface of the entire lung. Data are represented as scatterplot with overlaid Tukey box and whiskers graph (n = 14 per group), ∗∗p < 0.01 (Student's t test). (Right panel) Relative size distribution of lung tumors determined by sectioning of the entire lung, histological H&E staining, microscopic analysis, and measurement of tumor area in millions of pixel (px) using ImageJ software. The largest tumor area was quantified for nodules appearing in consecutive sections. Data are represented as percentage of all observed tumors in stacked bars (n ≥5 per group). (B) Flow cytometric quantification of frequency of the CD11b+ Ly6G + neutrophil population in the lung. Data are represented as individual values and mean ± SEM (n = 7 per group), ∗∗p < 0.01 (Student's t test). (C) Representative images of histological paraffin-embedded lung sections stained for S100A9 in brown to visualize neutrophils and counterstained with hematoxylin to label nuclei in blue. Scale bars, 100 μm. Dotted red line highlights tumor nodule margins. Insets show selected areas at higher magnification with black arrows pointing at neutrophils at the tumor-lung tissue interphase. See also Figure S2.

To test the general contribution of neutrophils to chemically induced carcinogenesis, we analyzed lung tumor onset triggered by urethane in neutropenic mice genetically lacking Csf3 (granulocyte colony-stimulating factor, Gcsf ko), the factor driving neutrophil maturation (Lieschke et al., 1994). Four months after the genotoxic insult, neutropenic mice develop fewer and smaller lung tumors compared with their control littermates, indicating that Gcsf ko mice are less sensitive to urethane-induced cancer (Figure 2A). Interestingly, in lungs harboring tumors, neutrophils were the only immune cell population increased in control mice compared with Gcsf ko mice (Figures 2B, 2C, and S2A–S2F). These results suggest an important role of neutrophils in the process of urethane-mediated induction of lung cancers.

### Neutrophils Are Directly Activated in Response to Urethane and Amplify the DNA Damage Response in Neighboring Cells *In Vitro*

Neutrophils have been previously reported to directly promote cancer cell growth and proliferation (Antonio et al., 2015; Houghton, 2010; Houghton et al., 2010; Wculek and Malanchi, 2015), which would be a relevant pro-tumorigenic activity during the second wave of their recruitment, the phase of cancer growth (Figure 1B). Moreover, neutrophils were described to indirectly promote colon cancer initiation by blocking adaptive immune responses against cancer cells (Katoh et al., 2013). Collectively, there is a bulk of knowledge that could explain a neutrophil-promoting activity during the neoplastic cell outgrowth in the lung (Houghton et al., 2010; Kargl et al., 2017). Therefore, we aimed to investigate whether neutrophils are involved in the process before this, specifically during the post-urethane initiation phase where their response could affect the predisposition to generate neoplastic cells in the tissue.

**Figure 3:**
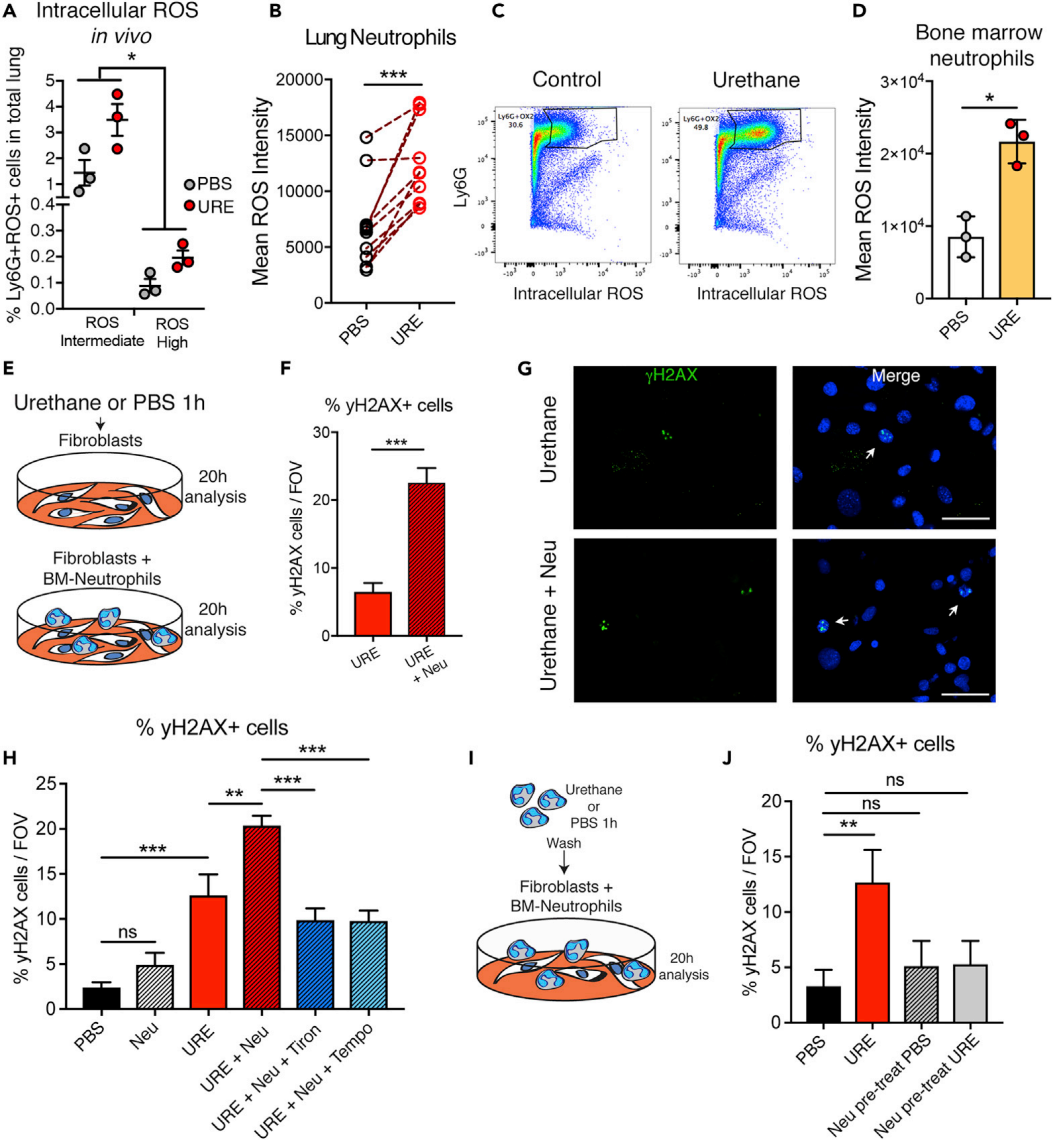
Neutrophils Induce a DNA Damage Response in Adjacent Cells upon Activation with Urethane *In Vitro* (A and B) Flow cytometric analysis of intracellular ROS activation in lung neutrophils. (A) Mice were exposed to urethane, and 24 h later, lungs were isolated and a single-cell suspension was generated. Intracellular ROS was measured using the DCFDA kit in Ly6G-positive cells. Quantification was done by flow cytometry on both the higher ROS and lower ROS pools of lung neutrophils. Data are represented as individual value and mean ± SEM, ∗p ≤ 0.05 (two-way ANOVA). (B) Freshly isolated total lung cells were prepared, and each lung cell suspension was exposed for 1 h either to urethane or to control PBS. Intracellular ROS levels of Ly6G+ neutrophils in each lung were determined using the DCFDA kit. Each dot represents ROS levels in neutrophils in the same lung upon either PBS or urethane (URE) treatment. Data are represented as individual paired values (n = 10), ∗∗∗p ≤ 0.001, (paired Student's t test). (C and D) Flow cytometric analysis of intracellular ROS activation in bone marrow (BM)-derived Ly6G+ neutrophils after *ex vivo* treatment with urethane or PBS (Control) for 1 h. Representative flow cytometric dot plot (C) and quantification (D) are shown. Data are represented as individual values and mean ± SEM (n = 3 BM neutrophil preparations), ∗p ≤ 0.05 (Student's t test). (E) Schematic of experimental design for data shown in (F and G). Normal fibroblasts were cultured with or without BM-derived neutrophils, urethane added for 1 h, washed, and analyzed after 20 h, with neutrophils excluded from the analysis. See also Figure S3A. (F and G) Quantification (F) of frequency of γH2AX foci-positive T-50 fibroblasts per 40× field of view (FOV) and (G) images of γH2AX-stained (green, arrows) fibroblasts after urethane treatment in the presence or absence of neutrophils (Neu). DAPI was used to identify nuclei (blue). Scale bars, 50 μm. Data are represented as mean ± SEM. One representative experiment of two is shown (n = 3 technical replicates, neutrophils mixed from 4 mice), ∗∗∗p ≤ 0.001 (Student's t test). (H) Quantification of frequency of γH2AX foci-positive T-50 fibroblasts untreated, treated with urethane, and treated with urethane and BM-derived neutrophils with or without the ROS scavenger Tiron or Tempo. Data are represented as mean ± SEM. (n = 3 technical replicates, neutrophils mixed from 4 mice), ∗∗p ≤ 0.01, ∗∗∗p ≤ 0.001 (Student's t test); ns, not significant. (I) Schematic of experimental design for data shown in (J). Normal fibroblasts were cultured with or without BM-derived neutrophils pretreated with urethane (Neu pre-treat URE) or PBS (Neu pre-treat PBS) for 1 h or directly treated with urethane for 1 h (URE) as positive control, washed, and analyzed after 20 h. (J) Quantification of frequency of γH2AX foci-positive T-50 fibroblasts. Data are represented as mean ± SEM (n = 3 technical replicates, neutrophils mixed from 4 mice), ∗∗p ≤ 0.01 (Student's t test). ns, not significant.

We first tested the direct reaction of neutrophils themselves to this genotoxic stimulus. We exposed lung to urethane and directly analyzed intracellular reactive oxygen species (ROS) in the Ly6G+ fraction as a marker of neutrophil activation (Wright et al., 2010). Neutrophils directly responded to urethane with an increase of intracellular ROS when lungs were exposed *in vivo* or when total lung cell suspensions were exposed *ex vivo* to urethane (Figures 3A and 3B). This effect could be recapitulated by directly exposing bone marrow-isolated neutrophils to urethane (Figures 3C and 3D). We next tested if this neutrophil response could influence the genotoxic effect of urethane on surrounding normal cells *in vitro*. We exposed normal fibroblasts to urethane in the presence or absence of bone marrow-derived neutrophils (Figure 3E). To detect the DNA damage response, cells were stained for histone H2AX phosphorylation (γH2AX) (Sharma et al., 2012). Indeed, when urethane exposure was performed in the presence of neutrophils, neighboring cells showed a higher percentage of cells undergoing a DNA damage response (Figures 3F and 3G). γH2AX staining was only monitored in normal fibroblasts, and the remaining neutrophils were excluded from the analysis (Figure S3A). Importantly, treatment with ROS inhibitors reverted the increase in DNA damage caused by neutrophil presence (Figure 3H), suggesting that this effect is ROS dependent.

Early studies have previously suggested that isolated neutrophils could spontaneously induce DNA damage in neighboring cells when co-cultured *in vitro* (Knaapen, et al., 1999). Notably, without urethane treatment, purified mouse neutrophils did not display any DNA damage activity (Figure 3H). As urethane treatment of neutrophils induces an intrinsic response and increases their ROS production (Figures 3C and 3D), we tested if urethane caused a stable effect in neutrophils, priming them for DNA damage activity in neighboring cells. Neutrophils were first exposed to urethane for the same length of time used in the co-culture (Figure 3E), to induce their activation and after co-cultured with stromal cells (Figure 3I). Interestingly, no significant increase in γH2AX positivity was detected in fibroblasts exposed to purified neutrophils previously activated by urethane (Figure 3J). This result suggests that urethane does not stably prime neutrophils for a DNA damage activity. In line with the observation that exposure to only urethane, but not only neutrophils, induces some DNA damage in normal fibroblasts (Figure 3H), neighboring cells apparently have to be exposed to urethane themselves, and neutrophil-mediated enhancement of DNA double-strand breaks in those neighboring cells, likely via ROS, only occurs at the time of urethane exposure. The absence of a persistent priming in neutrophil damaging activity in the tissue is reflected by the fact that urethane does not trigger disruption of tissue integrity as shown by collagen staining of lung sections as well as by podoplanin staining, an alveolar type I marker used to detect lung injury (McElroy and Kasper, 2004) (Figure S3B). The lack of tissue damage is also reflected in the absence of a broad inflammatory response (Figure S1).

### Neutrophils Amplify the DNA Damage Response in Lungs after Urethane Treatment *In Vivo*

Having observed neutrophils boosting the genotoxic power of urethane in normal surrounding cells in an *in vitro* co-culture assay, we next tested if neutrophils also affected urethane-dependent DNA damage induction *in vivo* within the lung tissue. To this end, we performed specific short-term neutrophil depletion using anti-Ly6G antibody (Daley et al., 2008; Wculek and Malanchi, 2015) during urethane treatment.

Considering the neutrophils' potential influence on immunological responses (Casbon et al., 2015; Coffelt et al., 2015; Spiegel et al., 2016), we first analyzed how other leukocytes in the lung react to urethane exposure in the context of neutrophil depletion. Treatment with anti-Ly6G antibody during urethane treatment and for the following week did not influence the urethane-induced reaction of other immune cell types (Figures S4A–S4G). These results indicate that the temporary changes in the presence of some types of leukocytes in the lung tissue early after urethane treatment are a direct response to the chemical insult and independent of a neutrophil-driven response.

**Figure 4:**
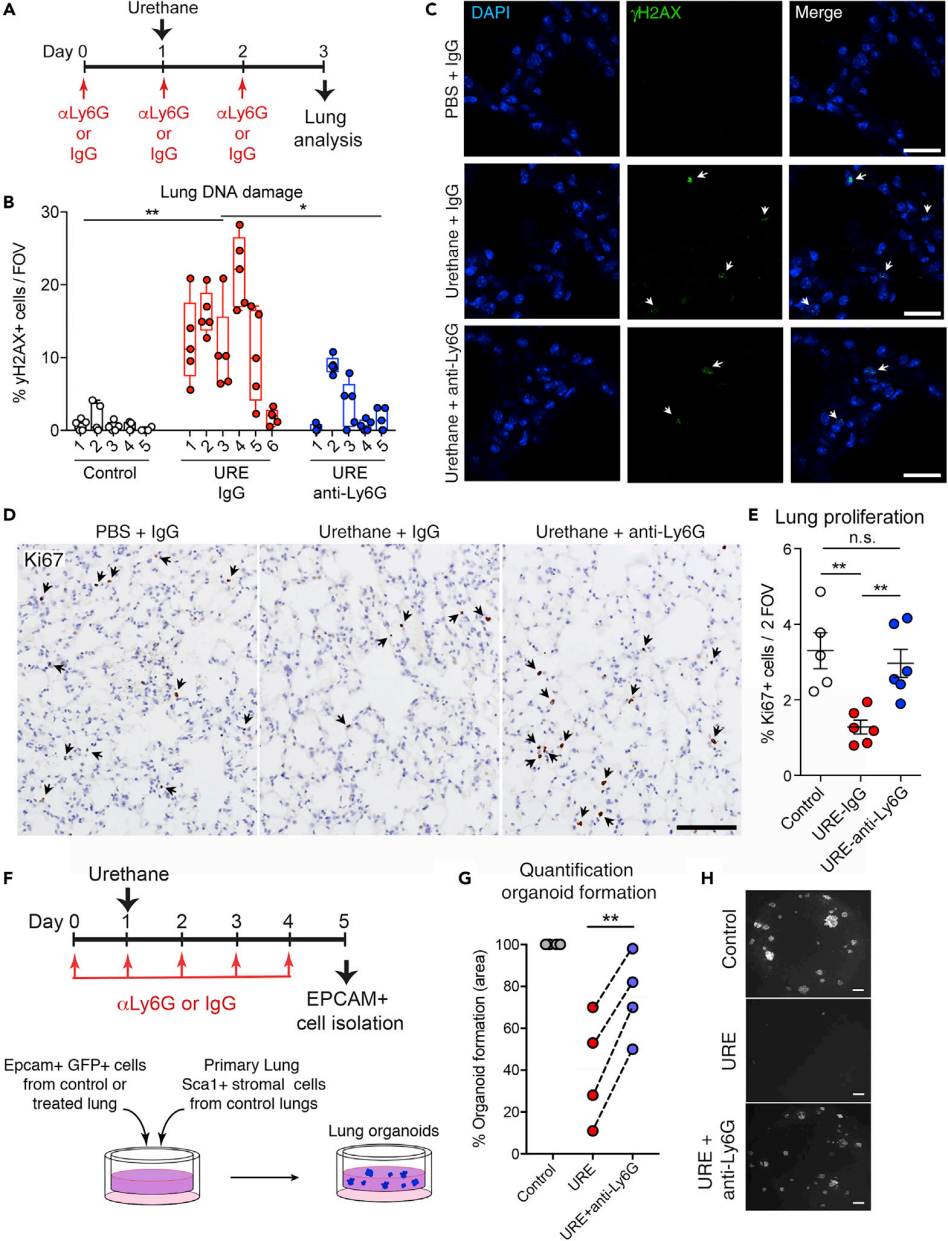
Neutrophil Depletion during Tumorigenic Initiation Reduces the DNA Damage Response (A) Schematic of experimental design for data shown in (B–E). Wild-type mice were treated daily with anti-Ly6G antibody or rat IgG isotype control in saline starting 1 day before intraperitoneal urethane (URE) or PBS (Control) administration and analyzed 3 days post-urethane. (B) Quantification of frequency of γH2AX+ cells in lung tissue sections of urethane (URE) or control PBS and Ly6G or IgG control antibody-treated mice to determine DNA damage. The percentage of γH2AX+ cells was calculated among total cells per 40× field of view (FOV). Numbers in x axis represent individual mice; circles represent percentage quantified in each animal (5 FOV were quantified per mouse). Data are represented as individual values and overlaid box plot that summarizes the total quantification of each group (n ≥ 5 mice per group), ∗p < 0.05, ∗∗p < 0.01 (Student's t test). (C) Representative images of data shown in (B). Scale bars, 25 μm. Arrows indicate γH2AX-stained cells (green) visualizing DNA double-strand breaks, and DAPI (blue) was used to stain nuclei. (D) Representative images of Ki67+ cells in lung tissue sections of urethane or control PBS and Ly6G or IgG control antibody-treated mice for quantification shown in (E). Scale bars, 100 μm. Arrows indicate Ki67-stained cells (brown) identifying proliferating cells, and hematoxylin (blue) was used to stain nuclei. (E) Quantification of frequency of Ki67+ cells among total cells per 20× FOV in lung tissue sections of urethane (URE) or control PBS and Ly6G or IgG control antibody-treated mice for determination of cell proliferation. Data are represented as individual values and mean ± SEM (n ≥ 5 mice per group, average of 2 random FOV calculated per lung tissue), ∗∗p < 0.01 (Student's t test), ns not significant. (F) (Upper panel) Schematic of experimental design for data shown in (G and H). eGFP-expressing mice were treated daily with anti-Ly6G antibody or rat IgG isotype control in saline starting 1 day before intraperitoneal urethane (URE) or PBS (Control) administration and EPCAM+ lung cells isolated 3 days post-urethane; (lower panel) schematic representation of co-culture organoid assay. (G) Quantification of organoids formed by EPCAM+ cells isolated from the different lungs. Each dot represents one flow cytometric sorting experiment. Data are shown as percentage of the PBS control condition in each experiment and are represented as individual experiments (n = 3 co-cultures per experiment), ∗∗p < 0.01 (paired Student's t test). (H) Representative fluorescent image of GFP+ organoids. Scale bar, 150 μm. See also Figures S3B and S4.

To test the influence of neutrophils on the lung tissue reaction to urethane, the DNA damage response was analyzed 3 days after urethane treatment. Mice were administered anti-Ly6G antibody to deplete neutrophils or isotype control antibody (IgG) before and for the following 3 days post-urethane (Figure 4A). Control lung tissue showed only sporadic DNA double-strand breaks (γH2AX foci), whereas urethane treatment triggered a DNA damage response in around 15% of lung cells. Strikingly, lungs depleted of neutrophils showed a strong reduction of γH2AX+ cell frequency (Figures 4B and 4C). Moreover, lung tissue 3 days post-urethane (when a high percentage of cells showed DNA double-strand breaks) also displayed a significant reduction in proliferating cells as measured by Ki67 staining. Remarkably, in line with the reduced DNA damage, the proliferation index of lung cells after urethane treatment in the absence of neutrophils did not decrease (Figures 4D and 4E). Together, these data suggest that neutrophils in the microenvironment change the lung tissue reaction to urethane.

To better test the magnitude of the urethane effect specifically in the lung alveolar type 2 cells (AT2), which were shown to be the cell of origin of lung adenocarcinoma (Xu et al., 2012), we assayed the fitness of lung epithelial cells upon urethane exposure in organoid reconstitution challenges. 3D co-culture systems have been developed to test *ex vivo* the intrinsic properties of different populations of lung epithelial cells. With the support of stroma, healthy lung epithelial cells will survive and grow in co-culture and form organoid structures in Matrigel (Kim et al., 2005; Lee et al., 2013; Ombrato et al., 2019). As shown in Figures 4B–4E, the genotoxic agent induces DNA damage in lung cells with the consequence of a short-term reduction of cellular proliferation. This might have an impact on the overall fitness of lung epithelial cells to maintain proliferation and survival capacity upon a challenge. To test the effect of the chemical specifically in epithelial cells, we tested their organoid formation ability (Figure 4F). When EPCAM+ lung cells, a cell pool that mainly contains AT2 cells, where isolated from lungs 5 days after urethane exposure, we observed a substantial reduction in organoid formation, which reflects a reduction of their ability to restart an organized “organ-like” growth *ex vivo* in a 3D environment (Figures 4G and 4H). This observation demonstrates the efficacy of the chemical insult urethane in targeting this cellular compartment. Remarkably, alveolar cells from neutrophil-depleted lungs treated with urethane show a greater capacity to establish organoids in co-culture (Figures 4G and 4H), confirming that absence of neutrophils in part protects them from the insult triggered by the chemical agent urethane.

Collectively, our data show that the presence of neutrophils increases the susceptibility of lung tissue to urethane genotoxicity and their absence reduces the effect specifically in the alveolar compartment.

### Neutrophil Recruitment Specifically during Urethane Exposure Rescues Tumorigenesis in Genetically Neutropenic Mice

**Figure 5:**
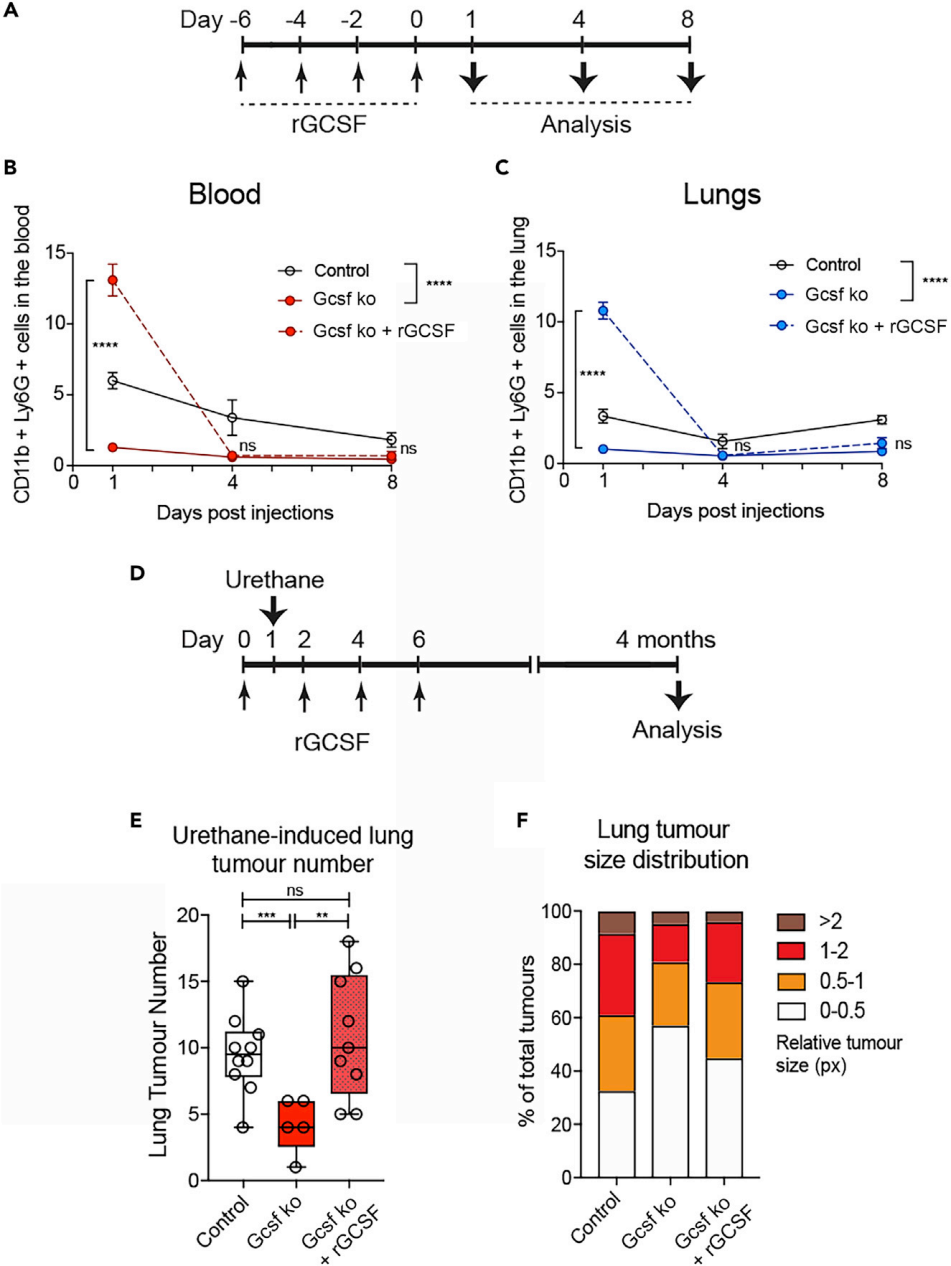
Mobilization of Neutrophils in Neutropenic Mice during Tumorigenic Initiation Restores Subsequent Cancer Formation (A) Experimental setup for data shown in (B and C) for neutrophil-induced mobilization using recombinant GCSF (rGCSF) in Gcsf ko mice. Gcsf ko mice were 4 times treated or not with rGCSF every 2 days and lungs analyzed 1, 4, or 8 days after the last rGCSF administration. Wild-type control mice were treated with PBS. (B and C) Flow cytometric quantification of frequency of the CD11b+ Ly6G+ neutrophil population among total alive cells in the blood (B) or lungs (C) in the indicated groups. Data are represented as mean ± SEM (n = 4 mice per group). ∗∗∗∗p < 0.0001; ns, not significant (Student's t test between Gcsf ko mice with or without rGCSF at each time point), ∗∗∗∗p < 0.0001 (two-way ANOVA between Control and Gcsf ko over time). (D) Experimental setup for data shown in (E and F). Gcsf ko mice were 4 times treated or not with rGCSF every 2 days and urethane injected 1 day after the first rGCSF administration. Wild-type control mice were treated with PBS. Lungs were analyzed 4 months thereafter. (E) Microscopic quantification of lung tumor numbers by histological sectioning and manual counting. Lung tumors appearing in consecutive sections were counted once only. Data are represented as scatterplot with overlaid Tukey box and whiskers graph (n = 9 Control and Gcsf ko + rGCSF; n = 5 Gcsf ko, two independent experiments), ∗∗p < 0.01, ∗∗∗p < 0.001 (Student's t test); ns, not significant. (F) Relative size distribution of lung tumors determined by sectioning of the entire lung, histological H&E staining, microscopic analysis, and measurement of tumor area in millions of pixel (px) using ImageJ software. The largest tumor area was quantified for nodules appearing in consecutive sections. Data are represented as percentage of all observed tumors in stacked bars. See also Figure S5.

Urethane carcinogenicity is tightly linked to its genotoxic effect, as extensive DNA damage is, in the longer term, the trigger of the genetic mutations causing cellular transformation. We have seen that neutrophils increase the sensitivity of the lung tissue to urethane-mediated insults; therefore, we tested the functional relevance of this initiation phase for long-term tumor formation. The reduced presence of neutrophils and the absence of their early recruitment in the lung throughout the entire period of tumor formation strongly inhibited the onset of lung cancer in genetically neutropenic (Gcsf ko) mice (Figure 2). Administration of exogenous recombinant GCSF (rGCSF) induces mobilization of neutrophils from the bone marrow in Gcsf ko mice, and their accumulation can be observed both in circulation and in the lung tissue (Figures 5A–5C). The level of neutrophils in circulation and in the lung tissue is increased by 3-fold by rGCSF administration in Gcsf ko mice; however, when rGCSF treatment is stopped, neutrophil levels rapidly decrease to their initial low levels within 4 days (Figures 5A–5C). To test the relevance of this temporally rescued neutrophil presence, specifically during urethane treatment, Gcsf ko mice where administered rGCSF starting 1 day before urethane exposure up to day 6 to maintain high neutrophil levels only during the first week post-urethane (Figure 5D). As shown (Figures 5A–5C), the termination of rGCSF treatment results in rapid return to severely reduced levels of neutrophils of Gcsf ko mice for the remaining 4 months of the tumorigenic process. Strikingly, rGCSF treatment of Gcsf ko mice completely rescued their lung tumor numbers 4 months after treatment compared with untreated Gcsf ko mice up to the tumor levels of control (Gcsf wild-type) mice (Figure 5E). When the tumor size distribution was analyzed, lung cancer nodules in rGCSF-treated Gcsf ko mice showed a reduction in large tumors compared with controls (Figures 5F and S5A). This suggests that the absence of neutrophils during the later cancer outgrowth phase (Figures 1B, 3 weeks to 4 months) might reduce tumor growth. Notably, Gcsf ko mice that were initially treated with rGCSF for 6 days (4 months before analysis) and that at endpoint harbor much more tumors compared with Gcsf ko mice that were never rGCSF treated also display a mild increase of neutrophils (Figure S5B). This highlights the potential of lung cancer to induce a local neutrophil recruitment even when their homeostatic levels in the circulation are severely reduced.

These results functionally uncouple early and late neutrophil activities during tumorigenesis and strongly suggest that the presence of lung neutrophils during carcinogenic urethane exposure enhances the overall tissue sensitivity to the oncogenic stress.

## Discussion

Genetic mutations causing permanent activation of oncogenes or inactivation of cancer suppressor genes are the primary cause of cancer, which is, hence, a genetic disease (Vogelstein and Kinzler, 2004). Particularly the right combination of genetic mutations needs to occur in the very same cell, and not every mutated tissue cell will have the potential to become a cancer-initiating cell. Indeed, a recent study directly proposes that what determine the organ cancer risk are oncogenic mutations combined with events triggering tissue regeneration (Zhu et al., 2016). Exposure to a variety of environmental carcinogens capable of inducing genetic mutations, such as chemical agents or UV light, are common events during life time and are the events that predispose a specific tissue to cancer. However, whether the specific cellular composition of a tissue contributes to tissue predisposition to cancer is currently not known.

In the present study, we investigated the contribution of neutrophils during the tissue response to spontaneous chemical carcinogenesis. To study neutrophil activity during tumor initiation, we used a model of lung cancer where the genotoxic agent urethane, a component of cigarette smoke, is used as the tumor-initiating agent. When mice are acutely exposed to urethane, lung tumors start developing after about 2 months, without the obvious induction of an active inflammation shortly after urethane exposure (Figures 1B and S1).

We here report that neutrophil presence within the tissue during the entire chemical carcinogenesis is essential for the efficiency of the process, as neutropenic mice show a strong reduction in urethane-induced lung cancers (Figure 2). Certainly, evidences are accumulating to describe the functions of neutrophils in promoting tumor growth, with the ability to generate an immune-suppressive environment being one of the more reported events (Coffelt et al., 2016). Indeed, we observed an acute reduction of lung T and NK cell frequencies 1-day post-urethane treatment, concomitantly with enhanced neutrophil presence, which normalizes within 1 week (Figures 1B, S1A, and S1B). However, no alteration in T or NK cells present in the lung of neutrophil-depleted mice compared with controls was detected early (1, 3, 5, and 7 days) after urethane administration (Figures S4B–S4C). Hence, a potential immunosuppressive function of neutrophils, which might contribute to urethane-mediated cancer initiation during the cancer cell outgrowth phase (after the third week), is unlikely to be present early after the genotoxic insult (within the first week) (Figure 1B). Interestingly, we found neutrophils to be the only inflammatory cell type transiently increasing their presence in the lung early after urethane administration *in vivo* (Figures 1A, 1B, and Figure S1), suggesting that they might directly react to urethane and potentially have a role in influencing generation of neoplastic cells in the tissue. Indeed, we show that the presence of neutrophils in the lung enhances the genotoxic effect of urethane exposure. Our data demonstrate that neutrophil depletion at the time of urethane treatment reduced the number of cells showing DNA double-strand breaks (Figures 4A–4C). We also observed a decrease in the overall proliferative activity of the tissue in consequence to urethane treatment (Figures 4D and 4E), which might be because cells subjected to DNA double-strand breaks activate checkpoint pathways that regulate DNA repair mechanisms and thereby arrest their proliferation (Branzei and Foiani, 2008). Accordingly, neutrophil-depleted urethane-treated lungs did not show a significant reduction in cellular proliferation (Figures 4D and 4E). To specifically test the effect of urethane in the lung alveolar type 2 cells (AT2), the cellular compartment giving rise to lung adenocarcinoma (Xu et al., 2012), we performed lung organoid assays. Healthy lung epithelial cells, co-cultured with supporting stroma cells in Matrigel, form organoid structures (Kim et al., 2005; Lee et al., 2013). Therefore, this 3D co-culture is a powerful system to test *ex vivo* the intrinsic potential of lung epithelial cells. As anticipated by the induction of DNA damage and the reduction in cellular proliferation (Figures 4A–4E), lung epithelial cells from urethane-treated lungs show a decrease in cellular fitness and a reduction of organoid formation (Figures 4F–4H), demonstrating the efficacy of the chemical insult to target this cellular compartment. However, when alveolar cells were isolated from neutrophil-depleted lungs treated with urethane, they show a greater capacity to establish organoids (Figures 4G and 4H), confirming that lung alveolar cells are specifically less affected by the chemical insult in the absence of neutrophils.

This effect is due to ROS production by neutrophils at the time of urethane exposure and amplifies its DNA-damaging effect on adjacent normal cells (Figure 3). Recently, in the context of chemically induced intestinal tumorigenesis, neutrophils were also shown to trigger genome-wide oxidative DNA damage via ROS release contributing to mutations and cancer growth (Canli et al., 2017). Notably, chemically induced intestinal tumorigenesis is characterized by an acute inflammatory response, consequent to an extended tissue damage, which breaks the intestinal barrier function inducing commensal bacterial infiltration. Conversely, we show that urethane systemically administrated intraperitoneally acutely affects the lung by increasing the level of DNA damage in lung cells without triggering a broad tissue damage or causing a great inflammatory response within 1 week post-urethane, as shown by the lack of immune infiltrates and the integrity of the lung architecture (Figures S1 and S3B). Subsequently, in line with the evidence that urethane and other anesthetics have slight, but significant, effects on the basal immune status of rats (Bette et al., 2004), urethane was reported to cause an inflammatory cytokine response in epithelial cells important for carcinogenesis. A previous study using a transgenic reporter showed activation of the inflammatory signaling nuclear factor (NF)-κB in lung epithelial cells from 2 weeks after urethane treatment demonstrating that the presence of cytokines such as tumor necrosis factor-α and interleukins (IL-10, IL-12, IL-6, etc.), promoted urethane-mediated lung carcinogenesis (Stathopoulos et al., 2007). Interestingly, the activation of NF-κB signaling in lung epithelial cells was subsequentially reported to be sustained by the presence of alveolar macrophages during the second and third weeks post-urethane as their depletion using clodronate liposomes prevented this response (Zaynagetdinov et al., 2011). Notably, these studies did not investigate the overall presence of inflammatory cells in the lung as alveolar macrophages were monitored by analyzing the bronchoalveolar lavage. Therefore, our study adds to the current knowledge that inflammatory cells and mediators are crucial for urethane-induced tumorigenesis in the lung, by revealing the early engagement of neutrophils in amplifying the genotoxicity of urethane. Importantly, our work highlights that early activities of neutrophils are mediated by a short-term ROS release specifically at the time of chemical exposure, which contributes to DNA damage without causing a tissue injury. Indeed urethane-primed neutrophils do not display any activity inducing DNA damage themselves (Figures 3H–3J). As neutrophil activity during sterile and non-sterile tissue injury is reported to enhance tissue damage (Del Fresno et al., 2018), it is important that mechanisms limit damaging neutrophil responses to preserve tissue integrity. Therefore, contrary to other chemically induced tumorigenesis such as the DMBA/TPA or AOM/DSS models, urethane tumorigenesis still relies on inflammatory cell-mediated activities, but in the absence of a broad inflammatory and damaging reaction in the tissue.

Importantly, this neutrophil-mediated enhancement of urethane genotoxicity leads to a long-term effect in lung cancer predisposition (Figure 5). This study uncouples the time-controlled neutrophil reaction inducing DNA damage via ROS as a consequence of urethane contamination in the lung from their tissue-damaging functions observed in the context of an extended inflammatory response. Our data show that lung neutrophil responses are finely controlled to preserve tissue homeostasis. Moreover, these data uncouple the early promoting effect of lung neutrophils on tumor initiation, where the increase in DNA damage likely enhances the chances of neoplastic transformation from normal to cancer cells, from their active cancer-promoting role during tumor outgrowth (Coffelt et al., 2016). Indeed, rescuing neutrophil recruitment to the lung of neutropenic mice specifically during the first week of urethane initiation recovers tumor incidence (Figure 5E), whereas the reduction in number of neutrophils in the lung seems to influence tumor growth and the size of tumors appear to be smaller compared with wild-type controls (Figures 5E and S5A).

Collectively, we show that the cellular composition of a tissue, specifically the presence of neutrophils in the lung, influences the tissue susceptibility to the carcinogenic agent urethane.

### Limitations of the Study

This study reports a synergistic effect of the exposure to the carcinogen urethane on epithelial cells and the activity of neutrophils specifically in lung tissue, where urethane induces cancers. If neutrophils in the lung are located in the perivascular niche, they are likely very exposed to urethane. Determining if those lung-specific responses of inflammatory cells trigger faster tumor onset in the lung than in other tissues, despite systemic urethane administration, will be interesting to evaluate in future dedicated studies. Also, it remains to be evaluated if neutrophils or epithelial cells in other tissues would respond to urethane similarly as in the lung.

### Resource Availability

#### Lead Contact

Further information and requests for resources and reagents should be directed to and will be fulfilled by the Lead Contact, Ilaria Malanchi (ilaria.malanchi@crick.ac.uk).

#### Materials Availability

This study did not generate new unique reagents.

#### Data and Code Availability

All data are included in the published article and the Supplemental Information files and any additional information will be available from the lead contact upon request.

## Methods

All methods can be found in the accompanying Transparent Methods supplemental file.

## Supplemental Information

Document S1. Transparent Methods and Figures S1–S5
