# Supplementary material for: Early Neutrophil Responses to Chemical Carcinogenesis Shape Long-Term Lung Cancer Susceptibility
Source: iScience. 2020 Jun 17;23(7):101277. doi: 10.1016/j.isci.2020.101277 (PMC7334367; doi:10.1016/j.isci.2020.101277)
Supplement: Document S1. Transparent Methods and Figures S1–S5 [file mmc1.pdf]

iScience, Volume *23*

Supplemental Information

Early Neutrophil Responses to Chemical Carcinogenesis Shape Long-Term Lung Cancer Susceptibility

Stefanie K. Wculek, Victoria L. Bridgeman, Freddie Peakman, and Ilaria Malanchi

Transparent Methods

Mice and treatments

Gcsf KO mice in mixed background (kind gift from Dr Joerg Huelsken (EPFL, Lausanne)) were used with their littermate controls for the experiment shown in Figure 2; Gcsf KO mice backcrossed to FVB/J (N10) and wild type FVB/J mice were used for the experiment shown in Figure 5. Transgenic mice expressing eGFP (Tg(CAG-EGFP)B5Nagy) under the chicken β-actin promoter in FVB/J background (kind gift from Dr Joerg Huelsken (EPFL, Lausanne)) were used for tissue (organoid assays). All other experiments were performed with FVB/J wild type mice. Sex-matched male and female mice were used and between 6-10 weeks of age. Breeding and all animal procedures were performed at our establishment in accordance with UK Home Office regulations under project license PPL/80/2531 and P83B37B3C. The NCRI Guidelines for the Welfare and Use of Animals in Cancer Research were strictly followed.

Urethane (Sigma, 1 mg/g mouse weight in PBS) was administered once by intra-peritoneal injection. Rat anti-Ly6G antibody (BioXcell, clone 1A8, 12.5 μg/mouse in PBS or saline (Daley et al., 2008)) or rat IgG isotype control (Cell Services Unit of The Crick Institute) was administered daily via intra-peritoneal injection. Recombinant GCSF protein (Novoprotein, C002, 5 μg/mouse in PBS) was administered sub-cutaneously to Gcsf KO and control mice every other day starting one day before and for the first week after urethane treatment, for a total of 4 injections.

Tumour burden evaluation

Macroscopic lung tumours were quantified by counting visible nodules on the lung surface using the Zeiss SteREO Lumar.V12 microscope. Lung tumour size distribution was determined by sectioning of the lung (100μm distance), histological H&E staining and microscopic analysis. The largest tumour area for each nodule was measured using ImageJ software applying the relevant pixel size of the image. No differences were observed between $Gcsf^{+/+}$ and $Gcsf^{+/-}$ mice and both merged into one control group in the experiments shown in Figure 2. In the rGCSF injection experiments using Gcsf ko mice in Figure 5E-F, all lung lobes were used for macroscopic quantification of tumour burden and tumour size determination by lung sectioning. However, for the latter, the smallest lung lobe of three random mice per group was instead used for lung neutrophil quantification by flow cytometry (shown in Figure 3G).

Histology

Lungs were fixed in 4% paraformaldehyde or NBF (normal buffered formalin), paraffin-embedded and 4μm sections stained with haematoxylin, eosin, Masson's Trichrome, S100A9 (clone 2B10) or Ki67 (clone SP6) antibodies using the VECTASTAIN® ABC kit (Vector Laboratories) and analysed using the Nikon Eclipse 90i light microscope and NIS-elements software. Podoplanin (clone 811) antibody staining was run on the VENTANA (Roche) staining robot.

Cell isolation and flow cytometry

Lung was dissected, minced, digested with Liberase (Roche) and DNaseI (Sigma) in HBSS while shaking and passed through a 100μm cell strainer (Wculek and Malanchi, 2015). Bone marrow cells obtained by crushing the femur and tibia and blood collected from the tail vein and Heparin or EDTA used as anti-coagulant. Single

cell suspensions were filtered, subjected to Red Blood Cell Lysis Solution (Miltenyi), washed and incubated with FcR Blocking Reagent (Miltenyi).

Bone marrow neutrophils were isolated by magnetic sorting using Ly6G antibody (BD, clone 1A8), microbeads and separation columns (all Miltenyi) with >90% purity. Lung epithelial cells and mesenchymal cells for organoid culture were isolated by magnetic sorting using EPCAM (CD326) antibody (eBioscience, clone G8.8) and Sca1 (Ly6A/E) antibody (Biolegend, clone D7), respectively, microbeads and separation columns (all Miltenyi) with ~75% purity. Mesenchymal cell preparations were first subjected to lineage depletion with CD45, CD31 and Ter119 (clones as outlined below).

CD45 (clone 30-F11), CD11b (clone M1/70), CD19 (clone 1D3), CD49b (clone DX5), Ly6G (clone 1A8), CD11c (clone HL3), CD3 (145-2C11), F4/80 (clone BM8), Ter119 (clone TER119), CD31 (clone 390) antibodies and DAPI (4,6-Diamidino-2-phenylindole dihydrochloride, Sigma) were used for flow cytometry with a LSRFortessa™ cell analyser, FACSDiva™ software (BD) and FlowJo software.

DNA damage analysis

The T-50 normal fibroblast cell line (kind gift from Dr Erik Sahai, (the Crick Institute)) was seeded in DMEM/F12 (Gibco) containing foetal calf serum, penicillin/streptomycin, Insulin/Transferrin/Selenium, Glutamine, HEPES, Heparin onto glass-bottom plates (Mattek) and bone marrow neutrophils (ratio 10:1) and urethane (1.4ng/ml) added. ROS inhibitors Tiron and Tempo (both Sigma) were used at 200µM. After 1 hr, cells were washed, incubated for 20 hrs at 37°C and fixed with 4% paraformaldehyde. Lungs were harvested 3 days after urethane treatment, fixed in 10% formalin, paraffin-embedded, 4µm sections cut and re-hydrated. Tissue and cells were blocked and permeabilised (Goat Serum, Bovine Serum Albumin, 0.1% Tween, all Sigma) before staining with yH2AX (clone JBW301) antibody, AlexaFluor488™ secondary antibody (Invitrogen) and DAPI and analysed with an inverted Zeiss 710™ Laser Scanning Confocal microscope and Zen 2010™ software.

ROS determination

Intracellular ROS (reactive oxygen species) was measured using the DCFDA-Cellular Reactive Oxygen Species Detection kit (Abcam, ab113851): total lung cell suspension or bone marrow-derived neutrophils were incubated with urethane (1.4ng/ml) and the DCFDA kit dye for 1 hr at 37°C, stained with Ly6G (BD, clone 1A8) antibody and analysed by flow cytometry.

Lung epithelial organoid culture

Lung organoid cultures were performed using EPCAM$^+$ cells isolated by magnetic cell sorting from constitutively eGFP expressing mice that had been pre-treated with Urethane or PBS and subjected to daily IgG or Ly6G treatment. Sca1$^+$ cells were isolated from untreated wild type FVB/NJ mice. 20,000 EPCAM$^+$ cells were cultured with 200,000 Sca1$^+$ cells in a total volume of 100µl of 50:50 Matrigel (Corning) in the top of 0.4µm pore 24-well transwell plates. 500µl of DMEM/F12 containing foetal calf serum, penicillin/streptomycin, Insulin/Transferrin/Selenium, Glutamine and sodium bicarbonate was added to the bottom of the transwell and changed every 2-3 days for 14 days. The plate was incubated at 37°C/10% CO_2. eGFP colonies were imaged after 14 days using the Zeiss SteREO Lumar.V12 microscope and their area was quantified using FIJI Software.

Statistical Analysis

Data are presented as mean ± standard error of the mean (SEM), individual values, stacked bars, scatter plot with Tukey box & whiskers bars, box plots or histograms and were analysed by unpaired or paired Student's t-test or Two-way ANOVA using GraphPad Prism version 7.

References

Daley, J.M., Thomay, A.A., Connolly, M.D., Reichner, J.S., and Albina, J.E. (2008). Use of Ly6G-specific monoclonal antibody to deplete neutrophils in mice. Journal of Leukocyte Biology 83, 64–70.

Wculek, S.K., and Malanchi, I. (2015). Neutrophils support lung colonization of metastasis initiating breast cancer cells. Nature 17, 413–417.

Supplemental Figures and legends

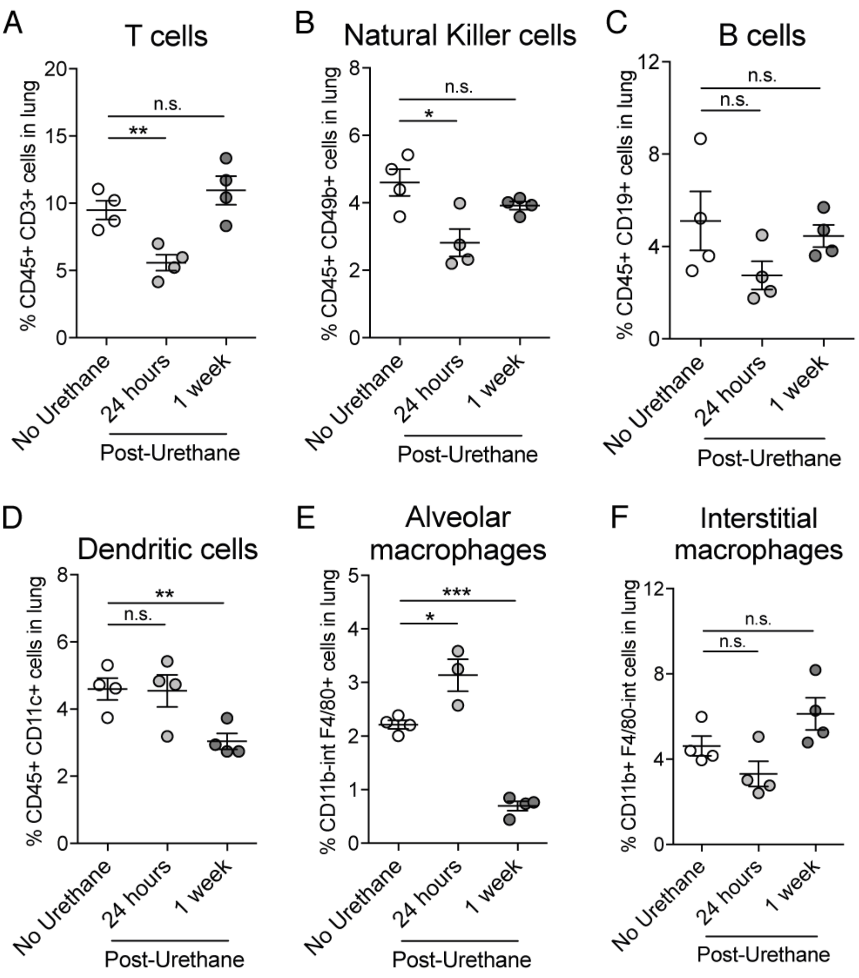

Figure S1. Immune cells infiltrating the lung upon urethane treatment. Related to Figure 1.

Flow cytometric quantification of frequency of immune cell populations in the lung of wild type mice at indicated times after urethane treatment. Data are represented as individual values and mean ± SEM (n=4 per time point, except for alveolar macrophages: n=3 at 24h time point), *P<0.05, **P<0.01, ***P<0.001 (Student's t-test), n.s. not significant: **A**, $CD45^+$ $CD3^+$ T cells, **B**, $CD45^+$ $CD49b^+$ natural killer cells, **C**, $CD45^+$ $CD19^+$ B cells, **D**, $CD45^+$ $CD11c^+$ dendritic cells, **E**, $CD11b^{low}$ $F4/80^+$ alveolar macrophages, **F**, $CD11b^+$ $F4/80^{low}$ interstitial macrophages.

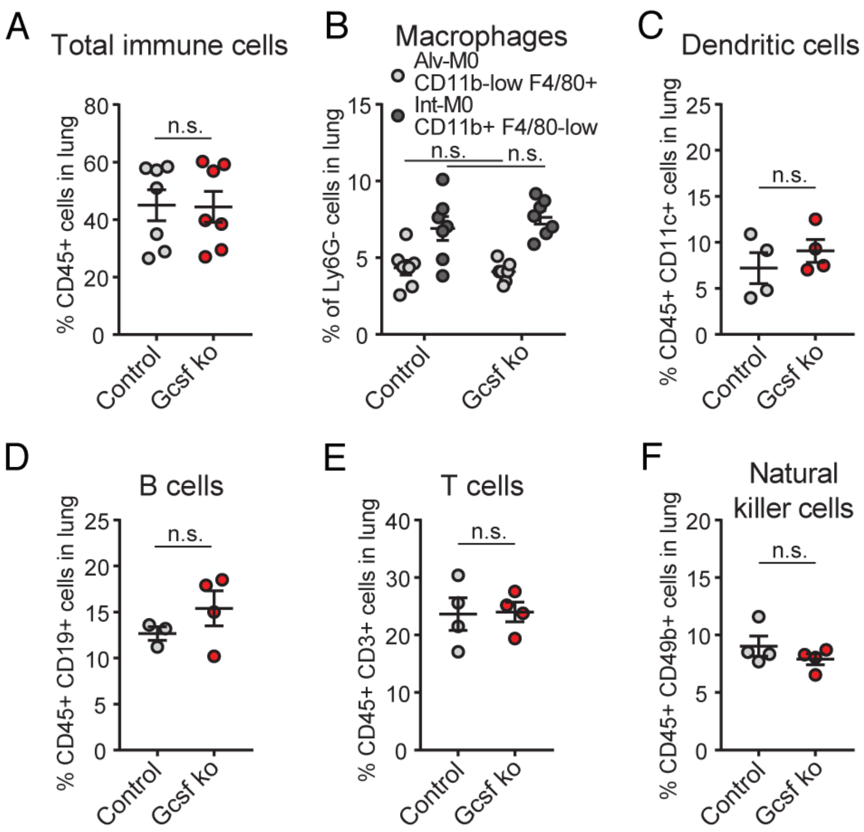

Figure S2. Lung leukocyte presence in urethane-induced lung tumour-bearing Gcsf ko mice. Related to Figure 2.

Flow cytometric quantification of frequency of immune cell populations in the lung of Csf3$^{+/+}$ or Csf3$^{+/-}$ (Control) and Csf3$^{-/-}$ (Gcsf ko) littermates 4 months after urethane injection. Data are represented as individual values and mean ± SEM, n.s. not significant (Student's t-test): **A**, CD45^{+} total immune cells (n=7 per group), **B**, CD11b^{low} F4/80^{+} alveolar macrophages (Alv-M0) and CD11b^{+} F4/80^{low} interstitial macrophages (Int-M0) (n=7 per group), **C**, CD45^{+} CD11c^{+} dendritic cells (n=4 per group), **D**, CD45^{+} CD19^{+} B cells (n=3-4 per group), **E**, CD45^{+} CD3^{+} T cells (n=4 per group) and **F**, CD45^{+} CD49b^{+} Natural killer cells (n=4 per group).

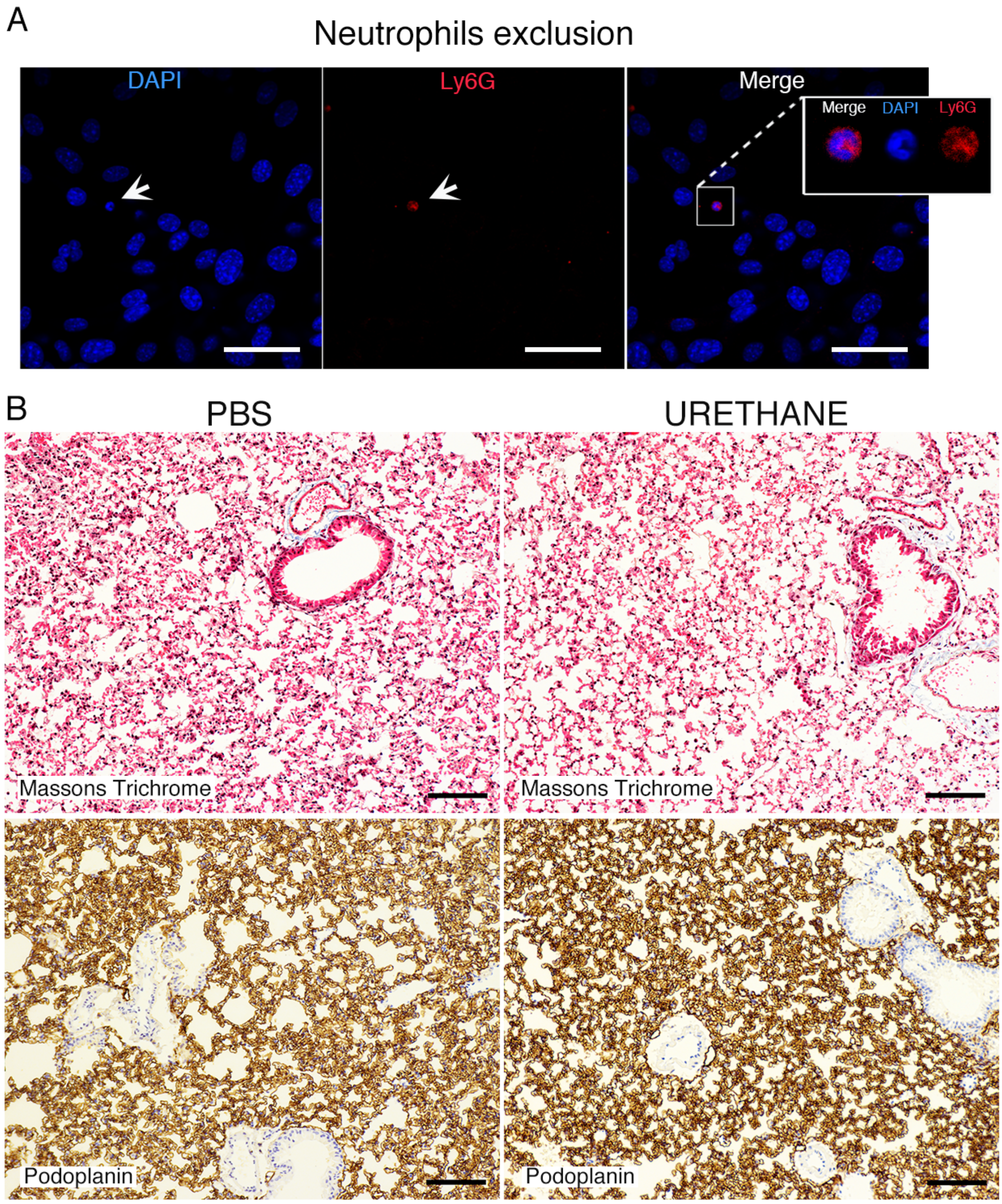

Figure S3. Lack of tissue damage in urethane-treated lungs. Related to Figure 3 and Figure 4.

A. Neutrophils exclusion from γH2AX^{+} cell quantification in the co-culture assays of Figure 3E-J. Firstly, the majority of neutrophils are removed by extensive washing. The few neutrophils remaining are identified based on the nuclear size and morphology and by the fluorescent Ly6G staining used for their magnetic purification. A representative fluorescent image of the small nucleus of a Ly6G stained neutrophil (arrow) compared to the fibroblast nuclei is shown. Insert highlights a selected area at higher magnification. Scale bar is 50µm µm. **B**, Representative histology of lung sections 3 days post-PBS or Urethane treatment stained with Massons Trichrome stain (Top Panel) or for Podoplanin (an alveolar type I marker used to detect lung injury) in brown and haematoxylin (to identify nuclei) in blue (Lower Panel). Scale bars are 50 µm.

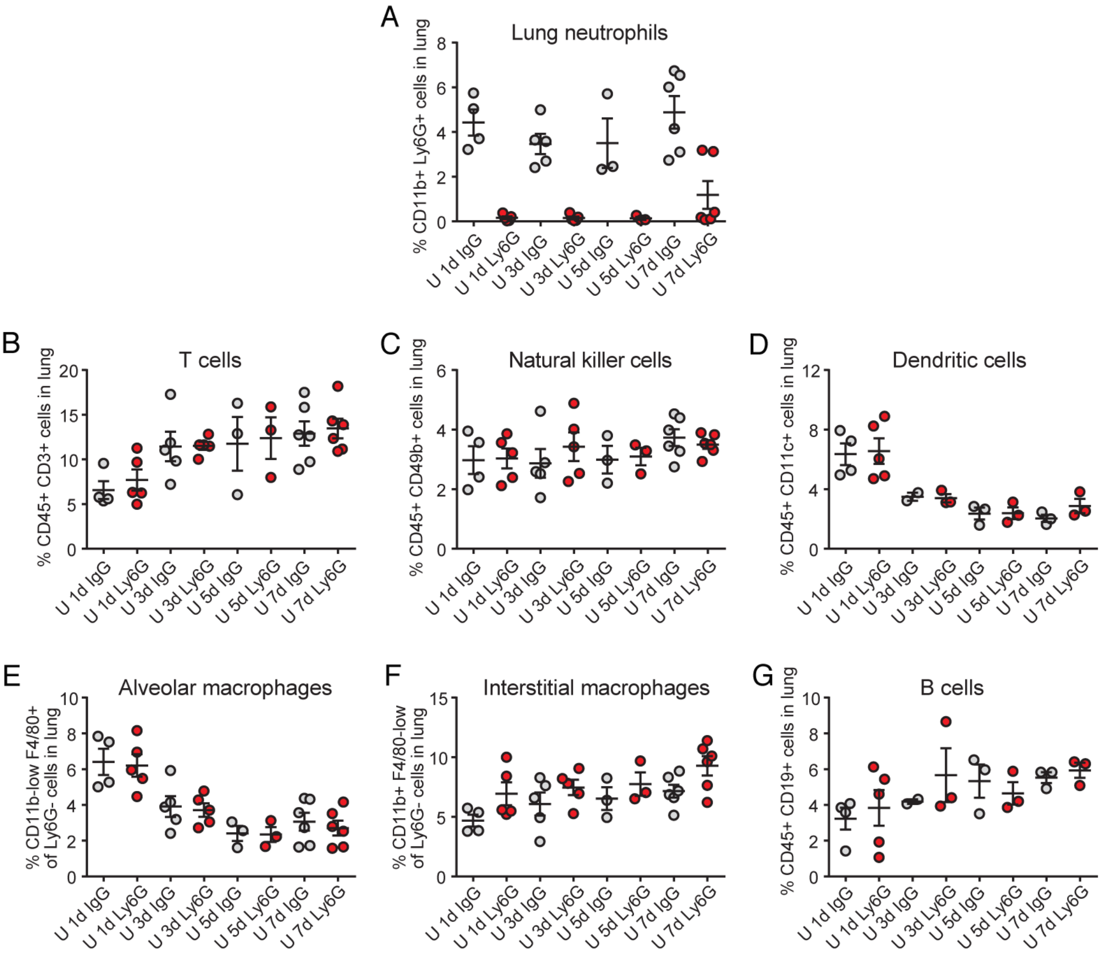

Figure S4. Lung leukocyte presence during initiation phase of urethane-mediated carcinogenesis in neutrophil-depleted mice. Related to Figure 4.

Flow cytometric quantification of frequency of immune cell populations in the lung of daily control IgG or neutrophil-depleting Ly6G antibody treated wild type mice 1, 3, 5 or 7 days (1d, 3d, 5d, 7d) after urethane (U) injection. Data are represented as individual values with mean ± SEM (n≥3 per condition, except for the U 3d IgG group in D and G were n=2): **A**, CD11b$^+$ Ly6G$^+$ neutrophils, **B**, CD45$^+$ CD3$^+$ T cells, **C**, CD45$^+$ CD49b$^+$ natural killer cells, **D**, CD45$^+$ CD11c$^+$ dendritic cells, **E**, CD11b^{low} F4/80$^+$ alveolar macrophages, **F**, CD11b$^+$ F4/80^{low} interstitial macrophages and **G**, CD45$^+$ CD19$^+$ B cells.

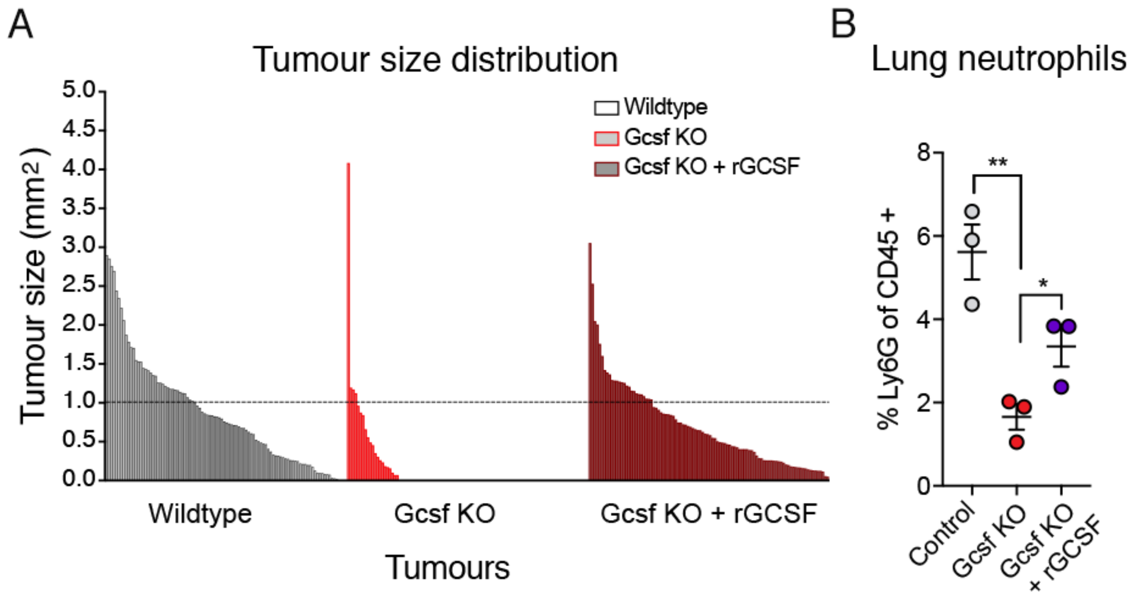

Figure S5. Neutrophil depletion during tumourigenic initiation modifies subsequent cancer formation. Related to Figure 5.

A, Histogram shows the size distribution histograms of all tumours generated 4 months after urethane treatment in wild type mice or Gcsf ko mice either untreated or treated with recombinant GCSF (rGCSF) only during the first week after urethane exposure (see Figure 5D). Each line represents one individual tumour. **B**, Flow cytometric quantification of frequency of the $CD11b^+$ $Ly6G^+$ neutrophil population in $CD45^+$ cells in lungs of control, Gcsf ko or rGCSF treated Gcsf ko mice 4 months after urethane treatment (n=3 mice per group). Data are represented as individual values and mean ± SEM. *P<0.05, **P<0.01 (Student's t-test).
